# Action of Mercury on the Liver

**Published:** 1871-09

**Authors:** 


### Action of Mercury on the Liver.

In the *Edinburgh Medical Journal* for April, Dr. T. R. Frazer presents the ablest and most exhaustive paper on the action of mercury on the liver that we have ever seen. He takes up and discusses *seriatim* the various doctrines in regard to the influence of mercury on the liver, viz.: "1. Mercury simply increases the flow of bile into the intestines. 2. It causes an increased formation of the bile by removing abnormal conditions that interfere with the secreting function of the liver. 3. It causes an increased formation of bile by an indirect action on the liver. 4. It causes an increased formation of bile by a direct and primary action on the liver. 5. Mercury has no cholagogue action whatever." After reviewing these propositions he concludes that we are entitled to maintain the *first* as true. The second, third, and fourth propositions may be regarded as elaborations of the first, advanced to explain the obvious effects on which it is founded. But in the present state of our knowledge of pathology and physiology it is impossible to prove that any one of these three is true. The fifth proposition maintained by Thudicum, Scott and Bennett has not been proved by them. In their experiments various disturbing agencies were present. Lesion of nerves, absorption of bile unmodified by digestion, the presence of inflamation and suppuration in the immediate vicinity of the liver, and imperfect digestion were all present, and must have exerted a modifying influence. The most important of these was the disturbance of digestion caused by interrupting the flow of the bile into the intestines. This alone would be sufficient to render inconclusive the experiments of these gentlemen.—*Michigan Med. Journal.*

